# A Case Report of Thrombotic Thrombocytopenic Purpura

**DOI:** 10.7759/cureus.94145

**Published:** 2025-10-08

**Authors:** Pragnesh D Patel, Devon Thorpe, Anita Sultan

**Affiliations:** 1 Internal Medicine, Overlook Medical Center, Summit, USA; 2 Hematology and Oncology, Overlook Medical Center, Summit, USA

**Keywords:** adamts13, rituximab, therapeutic plasma exchange, thrombotic microangiopathy, ttp

## Abstract

Thrombotic thrombocytopenic purpura (TTP) is a rare and potentially life-threatening condition characterized by fever, microangiopathic hemolytic anemia, thrombocytopenia, renal injury, and neurologic dysfunction. Until the advancements in treatment modalities in the late 20th century, TTP had carried a very high mortality risk. Treatment based on established guidelines has drastically reduced mortality rates, making early diagnosis and therapeutic intervention critically important. This case report discusses two patients diagnosed with TTP who had varying presentations, treatment courses, and recovery. The first case involves a 19-year-old male who initially presented with symptoms of a headache with some migraine-like features, which progressed to more severe neurological deficits before the diagnosis was made. The second case describes a 61-year-old female who presented with slurred speech but was seen to have TTP, which was refractory to initial management.

## Introduction

Thrombotic thrombocytopenic purpura (TTP) has an estimated worldwide incidence of 1.5-6 cases per one million adults annually, occurring more commonly in females with a 2:1 female-to-male ratio [1]. It is classified as a type of thrombotic microangiopathy (TMA), as the thromboses involve the capillaries and arterioles. TTP is further characterized by microangiopathic hemolytic anemia (MAHA), due to the platelet-rich microthrombi present in the small vessels, causing mechanical destruction of red blood cells, and thrombocytopenia, which can result in end-organ damage [2]. Although cases had been described as early as 1924, significant progress in understanding the pathophysiology was not made until the late 20th century.

The key pathogenic feature of TTP is the marked deficiency in ADAMTS13, a metalloprotease that is responsible for cleaving large multimers of von Willebrand factor (vWF). Without ADAMTS13, the multimers react with platelets in the microvasculature, leading to platelet aggregation, thrombosis, and subsequent hemolysis [3]. Diagnosis of TTP relies on high clinical suspicion and laboratory analysis. Signs of hemolytic anemia, such as the presence of schistocytes, elevated lactate dehydrogenase (LDH), or decreased haptoglobin, are often seen. The classic pentad of fever, thrombocytopenia, renal dysfunction, MAHA, and neurological abnormalities is frequently absent, only occurring concurrently in about 10% of cases. Nearly 60% of cases involve neurological symptoms involving headaches, seizures, coma, or stroke. Only about 10-27% of cases present with acute kidney injury, as renal manifestations more commonly present as isolated proteinuria or hematuria. Today, TTP has an annual incidence of one new case per million people and is twice as likely to occur in females as in males [4].

The treatment consists of steroids and therapeutic plasma exchange (TPE) in the acute setting, followed by immunosuppression thereafter. Though TTP can follow a relapsing clinical course, mortality rates have improved to 10-20% with appropriate management [4]. Although the diagnosis of TTP is well documented and standardized treatment protocols exist, real-world delays in diagnosis can occur due to atypical presentations or treatment refusal. We present two cases of TTP. The first case describes a patient who was initially diagnosed with a migraine and was later found to have TTP. The second case involves a patient who was accurately diagnosed at the time of presentation, but was not initially commenced on the optimal therapy.

## Case presentation

Case 1

A previously healthy 19-year-old male with no past medical or surgical history initially presented to the emergency department (ED) with a new-onset, severe left-sided headache accompanied by nausea, vomiting, and photophobia, and was diagnosed with a migraine headache at that time. His physical examination was unremarkable, and no laboratory investigations were done at the time. He declined getting a non-contrast CT scan and was discharged from the ED after clinical improvement with symptomatic treatment.

One week later, he presented to his neurologist with recurrent episodes of nausea and vomiting and a return of his headache, now accompanied by disorientation, aphasia, dysarthria, and right-sided weakness. He was referred to the ED due to concerns for possible underlying complex migraine; however, given the deterioration, a central nervous system infection or stroke was also considered in the differential diagnoses.

On his second presentation to the ED, he was now found to be jaundiced, with a left upper extremity hemiplegia, dysarthria, and expressive aphasia. Laboratory studies showed a white blood cell (WBC) count of 10.6 nL, hemoglobin level of 7.7 g/dL, platelet count of 27 nL, LDH level of 852 U/L, haptoglobin level of 73 mg/dL, and total bilirubin level of 2.9 mg/dL with a direct bilirubin of 0.4. Numerous schistocytes were seen on the peripheral blood smear, which was concerning for a hemolytic process such as MAHA. Admission labs, summarized in Table 1, showed profound thrombocytopenia, anemia, and elevated LDH, and decreased ADAMTS13 activity indicative of TTP. Improvement of these abnormal labs was seen by the end of the hospital stay.

**Table 1:** Summary of initial and final lab results WBC: white blood cell count; LDH: lactate dehydrogenase; ADAMTS13: a disintegrin and metalloproteinase with thrombospondin motifs 13

Test	Initial result (day 1)	Final result (day 9)	Reference range
WBC, nL	10.6	8.7	4.5 – 11
Hemoglobin, g/dL	7.7	10	14 – 17
Hematocrit, %	23.3	31.3	39 – 50
Platelets, nL	27	305	150 – 450
LDH, U/L	852	165	135 – 225
Haptoglobin, mg/dL	73	130	30 – 200
Creatinine, mg/dL	1.14	0.87	0.60 – 1.3
Total bilirubin, mg/dL	2.9 mg/dL	0.3	0 – 1.1
ADAMTS13 activity, %	<5	81	>66.8

Neuroimaging with non-contrast CT of the head was negative for hemorrhage, and CT angiograms of the head and neck were negative for stenosis. Hematology and critical care were consulted. Hematology assessed the patient as in the high-risk category for having a severely depleted ADAMTS13 level, given his PLASMIC score of 6, and he was transferred to the ICU for emergent plasmapheresis and high-dose steroids. Infectious disease was consulted, and he was empirically treated broadly with acyclovir, ceftriaxone, and vancomycin for possible meningitis; doxycycline and dapsone were given to cover for tick-borne disease.

Lumbar puncture was not pursued, given the patient's significant consumptive thrombocytopenia. After three days, his ADAMTS13 antibody activity returned as <5% (normal ≥70) with a Bethesda titer of 2.4 BU, which confirmed the suspected diagnosis. Given his improvement with plasmapheresis and steroids, and negative serology, infectious treatment was de-escalated and later discontinued. By day six of admission, his platelet count recovered to 203 nL. Throughout his admission, he received eight plasmapheresis treatments, was started on rituximab, and was initiated on a prolonged steroid taper. He was discharged after a nine-day hospitalization with complete resolution of all neurological deficits. He remains in remission six months post-discharge, with ADAMTS13 normalization.

Case 2

A 61-year-old female with no past medical or surgical history presented to the ED after waking up from a nap with slurred speech and altered mental status. Per the patient’s family, she had not seen a physician in 15 years and had been experiencing a generalized malaise for two weeks before admission, but declined medical attention. On the day of admission, the patient’s family reported that the patient had begun to act confused and agitated, which had prompted them to bring her to the emergency room.

Her initial labs were significant for normocytic anemia, elevated LDH, thrombocytopenia, hyperbilirubinemia, and a large amount of blood on urinalysis. Based on her initial labs, the patient had a PLASMIC score of 5, indicating intermediate risk for a severe ADAMTS13 deficiency. The patient’s mental status continued to worsen, raising concerns for TTP. Additional blood tests, such as reticulocyte count and ADAMTS13 activity, were also sent. Admission labs, summarized in Table 2, showed a profound thrombocytopenia, anemia, evidence of hemolysis, and normal renal function. Many of the abnormalities seen on admission labs improved upon discharge.

**Table 2:** Summary of initial and final laboratory results WBC: white blood cell count; LDH: lactate dehydrogenase; BU: Bethesda units

Test	Initial result (day 1)	Final result (day 11)	Reference range
WBC, nL	10.9	8.7	4.5 – 11
Hemoglobin, g/dL	7.8	10.2	14 – 17
Hematocrit, %	23.9	33.1	39 – 50
Platelets, nL	22	333	150 – 450
LDH, U/L	916	209	135 – 225
Haptoglobin, mg/dL	<10 mg/dL	Not collected	30 – 200
Creatinine, mg/dL	0.77	0.87	0.60 – 1.3
Total bilirubin, mg/dL	1.2	0.2	0 – 1.1
ADAMTS13 activity, %	<5	31	>66.8
ADAMTS13 Bethesda titer, BU	35.4	0.5	<0.5

Neurology was consulted for altered mental status, but there was low clinical suspicion of ischemic stroke, and CT angiogram of the head as well as CT arteriogram of the neck were both negative. Hematology was consulted for severe thrombocytopenia noted on bloodwork and concern for TTP. At this point, the patient’s family said it was against the patient’s wishes to receive blood products, and a shared decision was made to try steroid monotherapy with methylprednisolone 1000 mg administered intravenously (IV) for three days and reassess afterward. Rituximab was deferred as part of initial management per the patient's preference. At this time, the patient was also started on empiric antibiotic treatment for possible meningitis.

With steroid treatment, there was some improvement in the patient’s mental status. However, with minimal change in her hemoglobin and platelet count, the patient’s family became amenable to TPE. Her ADAMTS13 activity showed a value <5%, and TTP was confirmed. Once-daily plasma exchange with fresh frozen plasma (FFP) was given for a total of five sessions, with 12 total units of FFP being transfused. Her platelet count normalized for three days, and the patient was cleared for discharge with maintenance therapy of prednisone 40 mg daily and a plan to follow up her ADAMTS13 level as an outpatient for possible initiation of rituximab. At her follow-up visit, she was seen to have a decreased platelet count and was referred to the ED due to suspected refractory TTP. Her labs were significant for macrocytic anemia, thrombocytopenia, elevated LDH, and elevated lactic acid. There were no acute complaints during the second admission, and her mental status was near her baseline.

Hematology and nephrology agreed to continue with IV steroids during the hospital stay and restart plasma exchange therapy until a platelet goal of 150,000 /nL was reached (which was reached on day six). After her first plasma exchange session, the decision was made to also begin adjunctive weekly rituximab therapy. She received five total sessions of plasmapheresis and two doses of rituximab in the inpatient setting, with incremental improvement of her thrombocytopenia with each treatment. She was discharged with a steroid taper, weekly rituximab therapy, and instructions for close hematology follow-up. Time from symptom onset to definitive treatment was estimated to be more than two weeks. Table 3 presents a comparative summary analysis of both cases.

**Table 3:** Comparative summary analysis TPE: therapeutic plasma exchange; ADAMTS13 nadir: lowest enzyme level

Feature	Case 1	Case 2
Age, years	19	61
Sex	Male	Female
Initial symptoms	Headache, nausea, photophobia	Slurred speech, malaise
Delay to diagnosis, days	7	>14
Initial treatment	TPE + steroids	Steroids, delayed TPE
ADAMTS13 nadir, %	<5	<5
Use of rituximab	Early inpatient	Delayed (second admission)
Hospital stay length, days	9	11 + readmission
Outcome	Full recovery	Recurrence, ongoing treatment

## Discussion

Pathophysiology

TTP is characterized by visceral organ ischemia caused by platelet-rich microthrombi resulting from an ADAMTS13 deficiency <10% [4]. The ADAMTS13 enzyme usually functions to cleave vWF multimers, which prevents the formation of microthrombi. Under normal circumstances, ADAMTS13 circulates in a closed formation, whereas in acute episodes of TTP, it is seen in an open conformation, rendering the enzyme functionally ineffective. Deficiencies in this enzyme can occur congenitally due to biallelic mutations in the coding gene for ADAMTS13, as seen in Upshaw-Shulman syndrome, or can be acquired due to autoantibody formation against ADAMTS13, as is the case in the vast majority of diagnoses [4-5]. Presentations, diagnosis, and treatment remain largely the same between the aforementioned causes of TTP.

Diagnosis

While the classical pentad of symptoms, MAHA, and thrombocytopenia can lead to an increased suspicion for TTP, ADAMTS13 enzyme levels must also be obtained to support this diagnosis. This level is rarely available in emergent situations, and can often take three to seven days to get a result. The Harvard Research TMA Collaboration developed the PLASMIC score to assess the pretest probability of severe ADAMTS13 deficiency in patients with a TMA. This score is calculated by using platelet count, evidence of hemolysis, presence of active cancer, history of solid organ or stem cell transplant, MCV, INR, and creatinine [5-6]. PLASMIC scores ranging from 0 to 4 indicate low risk for TTP. Scores from 5 to 6 indicate intermediate risk and warrant ADAMTS13 testing. Scores greater than 6 are stratified as high risk and warrant expert consultation and TPE.

Treatment modalities

TTP had a mortality rate of 90% throughout most of the 20th century. It was not until 1991 that TPE, in conjunction with corticosteroids, started to become more widespread as a standardized treatment protocol for TTP [6]. The German protocol, which specifies therapeutic plasma exchange at a rate of 40-60 mL/kg daily until the platelet count is above 100,000 nL in patients with non-congenital TTP, has been credited with reducing mortality to 8% [7]. Adjuvant corticosteroids are also commonly used in recent treatment regimens, although data on their efficacy remain limited. It is proposed that the immunosuppressive effects of corticosteroids serve to inhibit ADAMTS13 autoantibodies, which leads to a quicker resolution of symptoms [8]. Both TPE and corticosteroids are considered first-line treatment options and are strongly recommended by the International Society on Thrombosis and Haemostasis (ISTH).

A more recent addition to the acute-phase treatment of TTP is caplacizumab, which now has a conditional recommendation per the ISTH. Caplacizumab is a nanobody that targets the A1 domain of vWF, preventing platelet binding and microthrombi formation. Caplacizumab was not administered in these cases, though emerging evidence supports its role both in reducing mortality in the acute setting and relapse risk as demonstrated in the TITAN and HERCULES trials [9]. The monoclonal CD20 antibody rituximab is another conditionally recommended treatment, which works by depleting the B-lymphocyte count, causing a decrease in ADAMTS13 antibody production [10]. It is used in conjunction with TPE in acute settings every three to four days, as well as weekly in the outpatient recovery setting. Though there are limited randomized controlled trials demonstrating its efficacy as an adjunct to TPE, excellent outcomes have been seen in patients with poor response to initial therapy.

Most commonly, rituximab is added when there is a suboptimal response to TPE and steroids alone or a delay in ADAMTS13 recovery. In our cases, caplacizumab was not administered, possibly due to limited institutional availability, clinical discretion, and patient preference [11]. The most common reason for delay in acute management of TTP has been seen to be diagnostic uncertainty. This can sometimes be due to a lack of schistocytes or thrombocytopenia appreciated on initial blood counts. One study showed that 27.8% of deaths associated with TTP could be linked to an initial delay in diagnosis [12].

## Conclusions

We discussed two presentations of TTP with varying outcomes. The first case showed the potential for a missed diagnosis due to an atypical presentation, causing a delay in treatment. The second case displayed a more timely diagnosis of TTP, but also involved some delay in treatment due to the patient’s wishes, despite a thorough risks/benefits discussion conducted by the hematology team. In both cases, the importance of early diagnosis via thorough clinical investigation is highlighted due to the possibility of rapid deterioration. The treatments these patients received varied in timing and may account for differences in recurrence, though other confounders, such as age and sex of the patients, most likely also played a role. These cases underscore that TTP requires urgent intervention regardless of presentation. Delays, whether from diagnostic uncertainty or patient refusal, may increase relapse risk. Early use of rituximab should be considered in refractory cases, and caplacizumab, if available, may further improve outcomes.
